# Development of a Melting Curve-Based Triple Eva Green Real-Time PCR Assay for Simultaneous Detection of Three Shrimp Pathogens

**DOI:** 10.3390/ani14040592

**Published:** 2024-02-11

**Authors:** Xuan Dong, Yujin Chen, Haoyu Lou, Guohao Wang, Chengyan Zhou, Liying Wang, Xuan Li, Jingfei Luo, Jie Huang

**Affiliations:** 1 State Key Laboratory of Mariculture Biobreeding and Sustainable Goods, Yellow Sea Fisheries Research Institute, Chinese Academy of Fishery Sciences, Laboratory for Marine Fisheries Science and Food Production Processes, Laoshan Laboratory, Key Laboratory of Maricultural Organism Disease Control, Ministry of Agriculture and Rural Affairs, Qingdao Key Laboratory of Mariculture Epidemiology and Biosecurity, Qingdao 266071, China; yjchen812@163.com (Y.C.); noyamelvin54@outlook.com (C.Z.); lixuan00213@163.com (X.L.); 19937593900@163.com (J.L.); huangjie@ysfri.ac.cn (J.H.); 2 Jiangsu Shufeng Aquatic Seed Industry Co., Ltd., Gaoyou 255654, China; 3 School of Fisheries and Life Science, Dalian Ocean University, Dalian 116023, China; 4 Network of Aquaculture Centres in Asia-Pacific, Bangkok 10090, Thailand

**Keywords:** aquaculture, shrimp pathogens, Eva Green, real-time PCR, detection

## Abstract

**Simple Summary:**

Shrimp aquaculture is an important industry due to its advantages of short production cycles and good economic benefits, but it faces challenges from diseases caused by tiny organisms that can lead to slow growth or large-scale shrimp deaths. Three such harmful pathogens are *Enterocytozoon hepatopenaei* (EHP), infectious hypodermal and hematopoietic necrosis virus (IHHNV), and Decapod iridescent virus 1 (DIV1). It is essential to quickly identify these organisms. In this study, we created a new test that can detect all three pathogens at once by using a melting curve-based Eva Green real-time PCR assay. We used this test on 190 shrimp samples from different regions in China and found that a significant number of shrimp were affected by these organisms. Our test is as accurate as the current standard test but faster because it looks for all three organisms simultaneously. This new method can help shrimp farmers identify diseases early, which is crucial for treating sick shrimp and preventing the spread of disease. This work is valuable because it offers a better way to keep shrimp healthy, which benefits the shrimp industry and supports aquaculture biosecurity.

**Abstract:**

Infections with *Enterocytozoon hepatopenaei* (EHP), infectious hypodermal and hematopoietic necrosis virus (IHHNV), and Decapod iridescent virus 1 (DIV1) pose significant challenges to the shrimp industry. Here, a melting curve-based triple real-time PCR assay based on the fluorescent dye Eva Green was established for the simultaneous detection of EHP, IHHNV, and DIV1. The assay showed high specificity, sensitivity, and reproducibility. A total of 190 clinical samples from Shandong, Jiangsu, Sichuan, Guangdong, and Hainan provinces in China were evaluated by the triple Eva Green real-time PCR assay. The positive rates of EHP, IHHNV, and DIV1 were 10.5%, 18.9%, and 44.2%, respectively. The samples were also evaluated by TaqMan qPCR assays for EHP, DIV1, and IHHNV, and the concordance rate was 100%. This illustrated that the newly developed triple Eva Green real-time PCR assay can provide an accurate method for the simultaneous detection of three shrimp pathogens.

## 1. Introduction

The aquaculture industry, particularly the shrimp sector, significantly contributes to global food security and economic development [1,2]. However, the industry faces substantial threats from various pathogens that can lead to severe diseases and substantial economic losses [3,4]. Among these, *Enterocytozoon hepatopenaei* (EHP), infectious hypodermal and hematopoietic necrosis virus (IHHNV), and Decapod iridescent virus 1 (DIV1) frequently encounter and are particularly notorious for their impact on shrimp health and productivity [5,6,7,8,9]. EHP is a pathogen that causes slow growth and reduced feed conversion efficiency in shrimp [5,10,11]. IHHNV is a well-known pathogen that can result in stunted growth and increased susceptibility to other diseases [6,7,12]. DIV1 is a newly identified virus that can cause systemic infection, leading to high mortality [8,9].

Rapid and accurate detection of these pathogens is crucial for the effective management and control of the diseases they cause [13,14,15,16,17,18]. Traditional diagnostic methods, such as histopathology and immunological assays, have limitations in terms of sensitivity, specificity, and the ability to detect multiple pathogens simultaneously. Molecular diagnostic techniques, particularly quantitative polymerase chain reaction (qPCR), have emerged as powerful tools for pathogen detection due to their high sensitivity and specificity [17,18,19]. Among these, real-time PCR assays with melting curve analysis using fluorescent dyes such as Eva Green offer advantages in terms of simplicity and cost-effectiveness while also allowing for the simultaneous detection of multiple targets in a single reaction [20,21].

In this context, the development of a melting curve-based triple real-time PCR assay using Eva Green fluorescent dye represents a significant advancement in the field of shrimp pathogen detection. This assay has the potential to streamline the detection process, enabling the simultaneous detection of EHP, IHHNV, and DIV1 with high specificity, sensitivity, and reproducibility. The present study aimed to validate the performance of this novel assay by analyzing clinical shrimp samples from various provinces in China and comparing the results with those obtained from the established TaqMan qPCR assay. The findings of this study are expected to contribute to the improvement of disease surveillance and control strategies in shrimp aquaculture, thereby enhancing the sustainability and productivity of this vital industry.

## 2. Materials and Methods

### 2.1. Samples and Nucleic Acids

All 190 clinical shrimp samples in this study were collected from Shandong, Jiangsu, Sichuan, Guangdong, and Hainan provinces in China. The positive nucleic acids for EHP, IHHNV, and DIV1, which were used for the establishment of the detection method, as well as the positive nucleic acids for white spot syndrome virus (WSSV), acute hepatopancreatic necrosis disease-causing *Vibrio parahaemolyticus* (*Vp*_AHPND_), infectious precocity virus (IPV), infectious myonecrosis virus (IMNV), yellow head virus genotype 8 (YHV-8), covert mortality nodavirus (CMNV), and *Macrobrachium rosenbergii* Golda virus (MrGV), which were used to assess the specificity of the detection method, were all obtained from shrimp samples collected by our laboratory in recent years.

### 2.2. Design of Primers

The primers were designed for the conserved regions of the three pathogens based on the spore wall protein 1 (SWP) gene sequence of EHP (GenBank accession No. KX258197.1), the parvovirus non-structural protein (NS1) gene sequence of IHHNV (GenBank accession No. JN616415.1), and the major capsid protein (MCP) gene sequence of DIV1 (GenBank accession No. KY681039.1). The primer design was performed using primer-BLAST (https://www.ncbi.nlm.nih.gov/tools/primer-blast/, accessed on 20 March 2022). The specificity of each primer set was assessed using the online BLAST tool (https://blast.ncbi.nlm.nih.gov/Blast.cgi?PROGRAM=blastn&PAGE_TYPE=BlastSearch&LINK_LOC=blasthome, accessed on 20 March 2022) to prevent cross-reactivity with non-target genes. The OligoAnalyzer™ tool was employed to analyze potential interactions among the three sets of primers (https://sg.idtdna.com/pages/tools/oligoanalyzer accessed on 21 March 2022). The tool was utilized to evaluate the propensity of individual sequences to form hairpin or dimer structures, as well as the potential for dimer formation between multiple sequences. A minimum of ten primer pairs were designed for each pathogen to facilitate subsequent selection. The refined primer designs were then synthesized by Sangon Biotech (Shanghai, China) Co., Ltd.

### 2.3. Construction of the Standard Plasmids

Three primer pairs, previously screened for specificity, were utilized to perform conventional PCR amplification to construct standard templates corresponding to the target sequences. The amplified products were resolved on a 1% agarose gel, and the resultant fragments were recovered using a gel extraction kit. These fragments were then ligated into the pUC-57 vector and transformed into competent cells. Positive clones were screened, and plasmids were extracted and sent to Beijing Tsingke Biotech (Beijing, China) Co., Ltd. for sequencing. The concentration of the recombinant plasmids (named pUC57-EHP, pUC57-IHHNV, and pUC57-DIV1, respectively) was determined using a NanoDrop 2000c spectrophotometer, with the procedure repeated three times to calculate an average concentration, which was then converted to copy numbers. The plasmid copy number was adjusted to 10^10^ copies/µL with ultrapure water and serially diluted to a range of 10^10^ to 10^0^ copies/µL.

### 2.4. Optimization of the Triple Eva Green Real-Time PCR Assay

All real-time PCR experiments were performed using the CFX96 system (Bio-Rad, Hercules, CA, USA). The optimal conditions were determined by altering the concentration of Mg^2+^ (1.5 mM, 2.0 mM, 2.5 mM, or 3 mM), each primer (0.3 μM, 0.4 μM, 0.5 μM, or 0.6 μM), and the annealing temperature (56–66 °C). Three parallel reactions were made for each condition by comparing the mean and standard deviation of the Ct values.

The real-time PCR systems contained: 0.1 μL 5 U/μL Accurate Taq HS DNA Polymerase (Accurate Biotechnology, Changsha, China), 2 μL 10× Taq PCR Buffer (Mg^2+^-free), 1 μL 50 mM MgCl_2_ Solution, 1.6 μL 12.5× dN(U)TP Mix, 0.2 μL 2 U/μL UNG enzyme, 1 μL 20× Eva Green^®^ Dye (Biotium, Fremont, CA, USA), 0.8 μL 10 μM each forward and reverse primer, 1 μL nucleic acid sample, and ddH_2_O to a final volume of 20 μL.

The following amplified parameters were obtained: an initial hold at 25 °C for 10 min, followed by a denaturation step at 95 °C for 2 min; this was succeeded by 40 cycles of 95 °C for 10 s and t °C (t = 56 °C, 58 °C, 60 °C, 62 °C, 64 °C, and 66 °C) for 20 s. The melting curve analysis was programmed to start with a hold at 95 °C for 15 s, followed by annealing at 64 °C for 1 min. Fluorescence data were collected starting at 68 °C and continued up to 90 °C to generate the melting curve.

### 2.5. Construction of Standard Curves

The optimized Eva Green real-time system was evaluated using a tenfold serial dilution of pUC57-EHP, pUC57-IHHNV, and pUC57-DIV1 plasmids, ranging from 1 × 10^10^ to 1 × 10^0^ copies/µL, along with a negative control. Each concentration was tested in triplicate. The standard curves were established by plotting the threshold cycle (Ct) values (*y*-axis) against the log of the initial DNA template concentrations (*x*-axis) using Bio-Rad CFX Maestro software (version 2.0).

### 2.6. Specificity Analysis

To assess the analytical specificity (ASp) of the triple Eva Green real-time PCR detection method, the DNA extracted from *Penaeus vannamei* samples infected with WSSV, *Vp*_AHPND_, IMNV, or CMNV; the cDNA synthesized using RNA from *Macrobrachium rosenbergii* samples infected with IPV, YHV-8, or MrGV; and the mixed seven types of pathogenic nucleic acids aforementioned were used as templates. Additionally, nucleic acid positives of EHP, IHHNV, and DIV1 were employed as positive control templates, total DNA from healthy *P. vannamei* was used as the negative control template, and nuclease-free water was used as the blank control template. Each template was tested in triplicate reactions.

### 2.7. Sensitivity Analysis

Plasmids pUC57-EHP, pUC57-IHHNV, and pUC57-DIV1, with concentrations ranging from 1 × 10^7^ to 1 × 10^0^ copies/µL, were prepared and utilized as templates to evaluate the analytical sensitivity (ASe) of the triple Eva Green real-time PCR detection method for each virus. The lowest detectable template copy number was determined based on the lower limit of fluorescence signal detection achievable by the instrument.

### 2.8. Repeatability Analysis

Different concentration gradients of the pUC57-EHP plasmid (1 × 10^9^ to 1 × 10^2^ copies/µL), pUC57-IHHNV plasmid (1 × 10^9^ to 1 × 10^2^ copies/µL), and pUC57-DIV1 plasmid (1 × 10^9^ to 1 × 10^2^ copies/µL) were selected as templates for the intra-assay and inter-assay repeatability experiments, with three replicates set for each concentration gradient. Intra-assay repeatability was assessed by repeating each concentration three times within the same batch of the triple Eva Green real-time PCR system, while inter-assay repeatability was evaluated by conducting the experiments on three different occasions for each concentration. The coefficient of variation (CV) was used to assess the repeatability of the triple Eva Green real-time quantitative PCR, defined as the percentage of the standard deviation of the Ct values obtained from repeated amplifications of each plasmid concentration gradient to the average Ct value.

The optimized triple real-time fluorescent quantitative PCR system and conditions were applied to the amplification with a Bio-Rad CFX96 real-time PCR system. Upon completion, Microsoft Excel was utilized for statistical analysis of the Ct values to analyze the repeatability of the triple Eva Green real-time fluorescent quantitative PCR method.

### 2.9. Detection of the Clinical Samples

The triple Eva Green real-time PCR detection method established in this study was applied to 190 shrimp samples collected from multiple provinces in China, including Shandong, Jiangsu, Sichuan, Guangdong, and Hainan. Concurrently, the detection of EHP was performed using the TaqMan qPCR method stipulated by Liu et al. [22], the World Organisation for Animal Health (WOAH)-recommended TaqMan qPCR method for IHHNV [23], and the Network of Aquaculture Centers in Asia-Pacific (NACA)-recommended TaqMan qPCR method for DIV1 established by Qiu et al. [19] (Table 1). The positivity rates of the two detection methodologies were compared.

## 3. Results

### 3.1. Primers Selection

**Figure 1:**
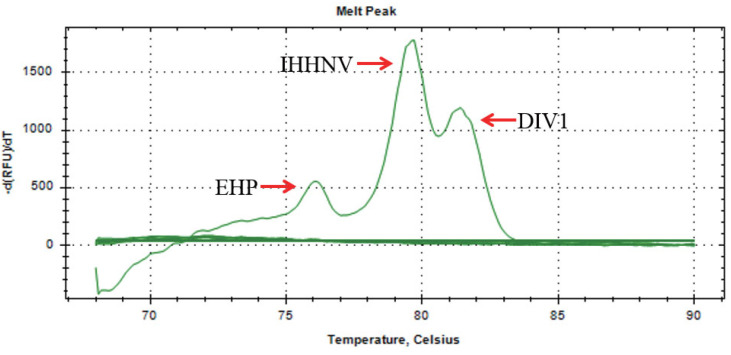
The melting curve of the triple Eva Green real-time PCR assay developed in this study.

During the screening of the 80 designed primer pairs, the first step involved selecting primers that could distinctly differentiate among the pathogens EHP, IHHNV, and DIV1 based on varying melting temperatures (Tm) (Figure 1). The second step entailed choosing primer pairs with high specificity, sensitivity, and stability from the initially screened sets corresponding to each pathogen. In this second phase of screening, the same sample was used to evaluate the primers, with preference given to those yielding lower cycle threshold (Ct) values and smaller standard deviations (SD) of Ct values in the real-time quantitative PCR assays. The primers finally selected to differentiate EHP, IHHNV, and DIV1 by the triple Eva Green Real-time PCR assay developed in this study are shown in Table 2.

### 3.2. Determination of the Optimal Reaction Conditions

After optimizing the concentrations of Mg^2+^ and each primer, the optimal composition of the triple Eva Green real-time PCR assay for a 20 μL reaction mixture was determined. The final concentration was set at 2.5 mM for Mg^2+^ (Table 3) and 0.4 μM for each primer (Table 4). Additionally, the optimized annealing temperature for the reaction was established at 64 °C (Table 5).

### 3.3. Generation of Standard Curves and Sensitivity Analysis

**Figure 2:**
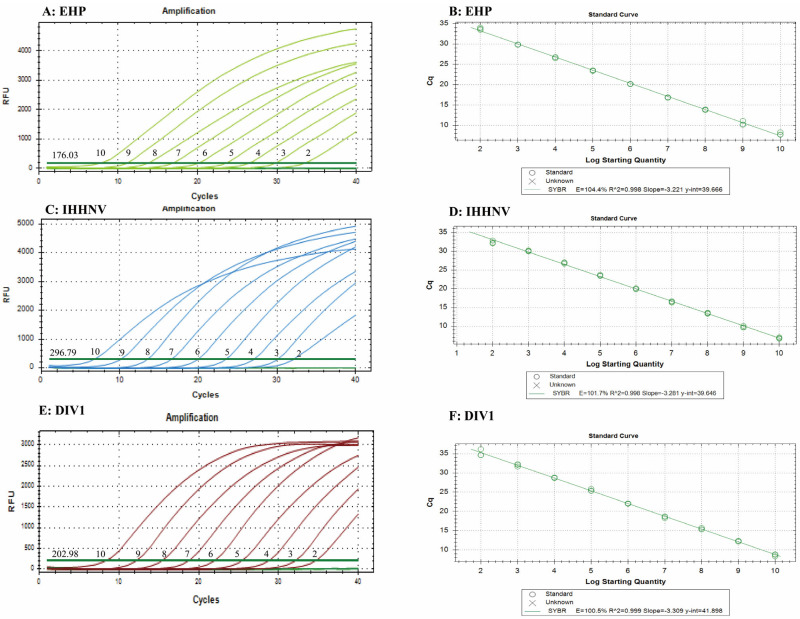
Amplification curve and standard curve of the triple Eva Green real-time PCR assay developed in this study. (**A**) Amplification curves of EHP; (**B**) standard curve of EHP; (**C**) amplification curves of IHHNV; (**D**) standard curve of IHHNV; (**E**) amplification curves of DIV1; (**F**) standard curve of DIV1. 1–10: 1 × 10^1^–1 × 10^10^ copies/reaction of the standard plasmids.

Based on the triple Eva Green real-time PCR reaction system and conditions determined in Section 3.2, plasmid standards for EHP, IHHNV, and DIV1 were serially diluted to create a gradient ranging from 10^10^ to 10^0^ copies/µL. These diluted plasmid standards were then used as templates for real-time PCR amplification. Standard curves were plotted by correlating the logarithm of the initial template concentration (X) with the Ct values (Y), as shown in Figure 2. The results indicated that a good linear relationship was observed when the concentrations of the three plasmid standards were between 10^10^ and 10^1^ copies/reaction. The amplification efficiencies for all three pathogens were within the optimal range (95–110%), and the correlation coefficients (R^2^) were equal to or greater than 0.998. The linear regression equations were as follows: for EHP (y = −3.221x + 39.666, R^2^ = 0.998, E = 104.4%), for IHHNV (y = −3.281x + 39.646, R^2^ = 0.998, E = 101.7%), and for DIV1 (y = −3.309x + 41.898, R^2^ = 0.999, E = 100.5%).

The results indicated that the assay could detect as few as 10^2^ copies/reaction of EHP, IHHNV, and DIV1, with corresponding Ct values of 33.60, 32.29, and 34.45, respectively. When the same amplification system was used to detect dilutions containing 10^1^ copies of EHP and DIV1, only two out of three replicates were positive. The detected Ct values for EHP were 36.22 and 36.51, while those for DIV1 were 35.76 and 37.68.

### 3.4. Specificity Analysis

**Figure 3:**
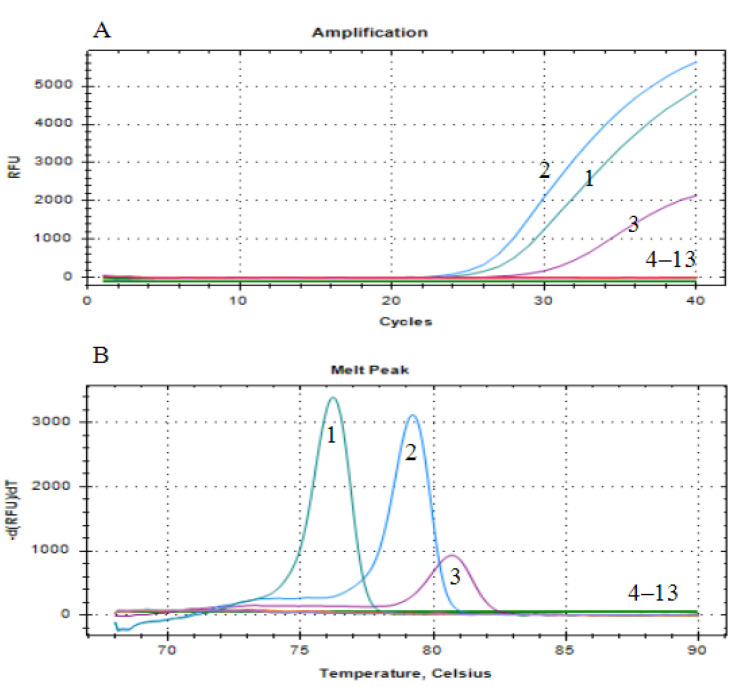
Specificity analysis of the triple Eva Green real-time PCR assay developed in this study. (**A**) Amplification curves; (**B**) melting curves. 1: EHP; 2: IHHNV; 3: DIV1; 4–13: WSSV, *Vp*_AHPND_, IPV, IMNV, YHV-8, CMNV, MrGV, mixed aforementioned seven types of pathogenic nucleic acids, blank control, and negative control.

Utilizing the triple Eva Green real-time PCR method established in Section 3.2, the amplification of positive DNA templates for EHP, IHHNV, and DIV1 yielded similar “S” shaped amplification curves. In contrast, no significant amplification curves were observed for other samples, including DNA from WSSV and *Vp*_AHPND_ and cDNA from IPV, IMNV, YHV-8, CMNV, and MrGV. The specificity of the triple Eva Green real-time quantitative PCR assay established in this investigation was evidenced by the absence of cross-reactivity with non-target pathogens (Figure 3).

### 3.5. Repeatability Analysis

To estimate the repeatability, plasmid standards ranging from 1.0 × 10^9^ to 1.0 × 10^2^ copies/μL were used as templates for real-time PCR amplification. The evaluation results indicated that within the initial template concentration range of 1.0 × 10^9^ to 1.0 × 10^2^ copies/μL, the triple Eva Green real-time PCR method exhibited commendable reproducibility. The coefficients of variation (CVs) of the intra-assay and inter-assay for the EHP plasmid were less than 1.63% and less than 2.61%, respectively (Table 6). The CVs of the intra-assay and inter-assay for the IHHNV plasmid were less than 2.04% and less than 2.31%, respectively (Table 6). The CVs of the intra-assay and inter-assay for the DIV1 plasmid were less than 1.31% and less than 1.90%, respectively (Table 6).

### 3.6. Detection of the Clinical Samples

A total of 190 shrimp samples collected from Shandong, Jiangsu, Nanjing, Sichuan, Guangdong, and Hainan provinces were tested. The results indicated that the Ct values for the samples that tested positive using the triple Eva Green real-time PCR assay ranged between 15.24 and 36.68. The triple Eva Green real-time PCR detection method successfully identified all positive samples that were detected by the singleplex TaqMan qPCR (Table 7), indicating 100% diagnostic sensitivity (DSe) and 100% diagnostic specificity (DSp). The positive detection rate for EHP was 10.5%; for IHHNV, it was 18.9%; and for DIV1, it was 44.2%. This assay also revealed the occurrence of co-infections with EHP and IHHNV, with a positive detection rate of 3.0%. Through the quantitative analysis of different concentrations of samples, the results showed that the Tm value ranges were 76.0–77.2 °C for EHP, 79.2–80.2 °C for IHHNV, and 81.2–82.0 °C for DIV1.

## 4. Discussion

The results from the current study underscore the utility of the triple Eva Green real-time PCR assay as a robust diagnostic tool for the simultaneous detection of EHP, IHHNV, and DIV1 in shrimp. In this paper, three kinds of probe-based real-time PCR detection methods were used in comparison [19,22,23]. The high concordance rate with the TaqMan qPCR assay, exhibiting 100% (Table 7), attests to the assay’s accuracy and reliability.

The detection limit of the EHP TaqMan qPCR method is as low as 4 × 10^1^ copies per reaction, 96 tests in <3 h [22]. A sensitivity test revealed that the DIV1 TaqMan qPCR assay could detect DIV1 DNA as low as 1.2 copies/reaction [19]. The IHHNV TaqMan qPCR assay has a detection limit of 10 copies [23]. Gao et al. (2023) established a duplex PCR for the detection of EHP and IHHNV, and the detection limit of the duplex PCR could reach 1.5 × 10^2^ copies for each pathogen [24]. A multiplex reverse transcription (RT)-PCR assay for the simultaneous detection of six viruses in shrimp was developed; however, the detection limits were not shown [25]. Liu et al. developed a multiplex PCR assay for the simultaneous detection of six viruses in shrimp in our laboratory [26]. The detection limits were 10^2^ copies for the detection of IHHNV, 10^3^ copies for Taura syndrome virus (TSV), 10^4^ copies for WSSV and hepatopancreatic parvovirus (HPV), and 10^5^ copies for *Baculovirus penaei* (BP) and IMNV [26]. The present results indicated that the triple Eva Green real-time PCR assay could detect as few as 10^2^ copies/reaction of EHP, IHHNV, and DIV1. Although this triple Eva Green real-time PCR assay is not more sensitive than the three singleplex TaqMan qPCR methods, it is on par with or even more sensitive than previously developed multiplex PCR methods. In addition, the triple Eva Green real-time PCR method can save more time/money than the single TaqMan qPCR methods and reduce the risk of aerosol contamination compared to conventional multiplex PCR detection methods because it does not require opening the lid to run the gel. More importantly, since the methodology relies on a melting curve, additional genetic markers for detecting other pathogens in shrimp can be integrated into the assay [27].

The sensitivity of the triple Eva Green real-time PCR assay, as demonstrated by the detection of positive samples across a wide range of Ct values (15.24–36.68), suggests that the assay is capable of identifying infections at various stages, including early and low-level infections that might be missed by less sensitive methods. This is crucial for implementing effective disease control measures and preventing the spread of infections within and between shrimp farms.

The high specificity of the assay is evidenced by the absence of cross-reactivity with non-target shrimp pathogens and shrimp tissues. In China, *P. vannamei* is the largest farmed penaeid shrimp species, which has been frequently affected by these three pathogens and some other pathogens [28,29]. In recent years, the impact of DIV1 on *M. rosenbergii* has been increasing [9]. The specificity of a sensitive detection method targeting three pathogens is essential to ensure the accuracy of the diagnostic results, especially when facing the challenge of coinfection with various pathogens in farmed shrimp. We were satisfied with the ASp of the triple method, which we assessed using nucleic acid positives of WSSV, *Vp*_AHPND_, IMNV, and CMNV from *P. vannamei* samples, as well as IPV, YHV-8, and MrGV from *M. rosenbergii* samples. Furthermore, we achieved 100% DSp and 100% DSe with 190 clinical samples. Despite these promising results, a more rigorous assessment would require evaluating a wider range of host and pathogen samples.

The observed high positive rates of EHP, IHHNV, and DIV1 in the clinical samples from various provinces in this study might be attributed to the sampling strategy specifically targeting diseased samples. The detection of co-infections, as indicated by the presence of EHP and IHHNV in the same samples, highlights the complexity of disease dynamics in shrimp populations and the necessity for comprehensive diagnostic approaches.

Through quantitative analysis of various sample concentrations, our results revealed distinct Tm value ranges for each pathogen. Specifically, the Tm value range for EHP was determined to be 76.0–77.2 °C, while the Tm value range for IHHNV was found to be 79.2–80.2 °C. Additionally, the Tm value range for DIV1 was observed to be 81.2–82.0 °C. These findings highlight the discriminatory power of our melting curve-based triple Eva Green real-time PCR assay in accurately identifying and differentiating these three shrimp pathogens.

In conclusion, the melting curve-based triple Eva Green real-time PCR assay represents a significant advancement in the molecular diagnostics of shrimp pathogens. The assay’s performance in this study suggests that it could be widely adopted for routine screening and the rapid response to disease outbreaks in the shrimp industry. Future studies should focus on the assay’s applications in different aquaculture systems and its integration into broader pathogen monitoring and biosecurity frameworks.

## 5. Conclusions

The newly developed triple Eva Green real-time PCR assay has proven to be a highly specific, sensitive, and reproducible diagnostic tool for the concurrent detection of EHP, IHHNV, and DIV1 in shrimp. The assay’s validation on a substantial number of clinical samples from diverse geographic regions in China, coupled with a concordance rate of 100% when compared to the TaqMan qPCR assay, underscores its potential as a reliable method for pathogen surveillance and disease management in the shrimp aquaculture industry.

## Figures and Tables

**Table 1:** TaqMan qPCR primers and probes for EHP, IHHNV, and DIV1 in this study.

Primer	Sequence (5′–3′)	References
EHP-F157	AGTAAACTATGCCGACAA	[22]
EHP-R157	AATTAAGCAGCACAATCC	
EHP-TaqMan probe	FAM-TCCTGGTAGTGTCCTTCCGT-TAMRA	
IHHNV-1608F	TACTCCGGACACCCAACCA	[23]
IHHNV-1688R	GGCTCTGGCAGCAAAGGTAA	
IHHNV-TaqMan probe	FAM-ACCAGACATAGAGCTACAATCCTCGCCTATTTG-TAMRA	
DIV1-142F	AATCCATGCAAGGTTCCTCAGG	[19]
DIV1-142R	CAATCAACATGTCGCGGTGAAC	
DIV1-TaqMan probe	FAM-CCATACGTGCTCGCTCGGCTTCGG-TAMRA	

**Table 2:** Primers that were used to differentiate EHP, IHHNV, and DIV1 in this study.

Primer	Sequence (5′–3′)	Amplicon Size (bps)	Tm (°C)
EHP-F	GCCGAGTTTGGCGGCACAATTCTCA	117	76.0
EHP-R	CAGGTCCTACAAATGCTGTGTCTGTGTAA		
IHHNV-F	GCACCGCTACAAACAGCTATGGACCCG	103	79.7
IHHNV-R	GCGCCGTACAGTTGGCTTTGGTTTGAC		
DIV1-F	GTTGGTGCGGCCCACAATGG	79	81.4
DIV1-R	GTGAGGGGGCAACGGCGATA		

**Table 3:** Mg^2+^ concentration optimization results.

Sample Ct Values/Mg^2+^ Concentration	1.5 mM	2.0 mM	2.5 mM	3 mM
EHP-mean of Ct values	27.03	26.69	27.02	27.26
EHP-standard deviation of Ct values	0.10	0.50	0.17	0.16
IHHNV-mean of Ct values	30.75	27.01	26.92	27.34
IHHNV-standard deviation of Ct values	1.18	0.03	0.15	0.15
DIV1-mean Ct Value	27.61	27.83	27.64	28.29
DIV1-standard deviation of Ct values	0.26	0.08	0.22	0.01

**Table 4:** Primer concentration optimization results.

Sample Ct Values/Primer Concentration	0.3 μM	0.4 μM	0.5 μM	0.6 μM
EHP-mean of Ct values	26.70	26.69	27.14	27.11
EHP-standard deviation of Ct values	0.14	0.04	0.14	0.07
IHHNV-mean of Ct values	——	26.74	27.58	27.39
IHHNV-standard deviation of Ct values	——	0.14	0.35	0.12
DIV1-mean Ct Value	——	27.48	27.99	30.02
DIV1-standard deviation of Ct values	——	0.12	0.40	0.81

—— Indicates that no result was generated.

**Table 5:** Annealing temperature optimization results.

Sample Ct Values/Annealing Temperature	66 °C	64 °C	62 °C	60 °C	58 °C	56 °C
EHP-mean of Ct values	27.58	27.33	27.40	26.77	26.52	27.5
EHP-standard deviation of Ct values	0.14	0.19	0.06	0.60	0.59	0.15
IHHNV-mean of Ct values	31.23	27.69	27.56	26.89	27.18	26.37
IHHNV-standard deviation of Ct values	0.14	0.21	0.04	0.31	0.30	1.46
DIV1-mean Ct Value	28.24	27.93	28.37	27.86	28.32	28.47
DIV1-standard deviation of Ct values	0.17	0.13	0.07	0.21	0.11	0.14

**Table 6:** Intra-assay and inter-assay variability of the triple Eva Green real-time PCR assay.

Plasmid	Concentration (Copies/μL)	Intra-Assay Ct			Inter-Assay Ct		
		$\bar{x}$	SD	CV (%)	$\bar{x}$	SD	CV (%)
pUC57-EHP	1.0 × 10^9^	10.20	0.17	1.63	10.23	0.12	1.20
	1.0 × 10^8^	13.74	0.09	0.63	13.63	0.29	2.13
	1.0 × 10^7^	16.72	0.05	0.27	16.35	0.41	2.54
	1.0 × 10^6^	20.08	0.04	0.20	19.63	0.51	2.61
	1.0 × 10^5^	23.35	0.09	0.36	22.91	0.50	2.17
	1.0 × 10^4^	26.53	0.15	0.55	26.02	0.57	2.20
	1.0 × 10^3^	29.70	0.02	0.05	29.27	0.48	1.62
	1.0 × 10^2^	33.60	0.25	0.74	33.13	0.57	1.72
pUC57-IHHNV	1.0 × 10^9^	9.81	0.20	2.04	9.89	0.23	2.31
	1.0 × 10^8^	13.38	0.14	1.05	13.32	0.20	1.53
	1.0 × 10^7^	16.41	0.18	1.07	16.35	0.24	1.47
	1.0 × 10^6^	19.89	0.16	0.81	19.90	0.18	0.90
	1.0 × 10^5^	23.47	0.15	0.64	23.47	0.11	0.47
	1.0 × 10^4^	26.78	0.19	0.71	26.67	0.21	0.79
	1.0 × 10^3^	29.98	0.17	0.55	30.05	0.18	0.60
	1.0 × 10^2^	32.29	0.39	1.22	32.62	0.47	1.44
pUC57-DIV1	1.0 × 10^9^	12.01	0.15	1.21	11.94	0.14	1.14
	1.0 × 10^8^	15.18	0.20	1.31	15.17	0.29	1.90
	1.0 × 10^7^	18.24	0.21	1.18	18.03	0.31	1.75
	1.0 × 10^6^	21.67	0.08	0.38	21.44	0.28	1.30
	1.0 × 10^5^	25.21	0.23	0.90	25.02	0.27	1.07
	1.0 × 10^4^	28.39	0.05	0.17	28.31	0.20	0.71
	1.0 × 10^3^	31.68	0.34	1.08	31.50	0.54	1.70
	1.0 × 10^2^	34.45	0.32	0,92	34.03	0.55	1.61

**Table 7:** The real-time PCR detection results in clinical samples.

Pathogens	Total Clinical Samples	Positive Rate	
		Triple Eva Green qPCR	TaqMan qPCR
EHP	190	10.5% (13/190)	10.5% (13/190)
IHHNV	190	18.9% (29/190)	18.9% (29/190)
DIV1	190	44.2% (86/190)	44.2% (86/190)
EHP and IHHNVco-infection	190	3.0% (7/190)	3.0% (7/190)

## Data Availability

Data are contained within the article.
